# An update on the network theory of epilepsy

**DOI:** 10.3389/fnetp.2026.1442019

**Published:** 2026-05-11

**Authors:** Hitten P. Zaveri, Steven M. Pincus, Robert B. Duckrow, Dennis D. Spencer

**Affiliations:** 1 Departments of Neurology, Yale University, New Haven, CT, United States; 2 Guilford, CT, United States; 3 Departments of Neurosurgery, Yale University, New Haven, CT, United States

**Keywords:** brain networks, connectivity, epilepsy surgery outcome, local functional connectivity, network theory of epilepsy, seizure onset area

## Abstract

We provide an understanding of the network theory of epilepsy based on an interpretation of temporal, spatial, spectral and connectivity evaluations of background intracranial electroencephalogram (EEG) recordings. The background intracranial EEG recordings were acquired from patients with medically intractable epilepsy undergoing evaluation for epilepsy surgery. We argue that our results suggest, in the main, the presence of a two-node network with one of the nodes being the seizure onset area, and the second the thalamus. We hypothesize seizures arise from the interaction of these two nodes under a specific set of circumstances.

## 1 Introduction

We provide a brief overview of the two main theories of epilepsy. This is followed by an update of results published by our group, and a discussion on how they could inform a two-node theory of epilepsy. This material has been presented in part at Grand Rounds at the George Washington University School of Medicine and Health Sciences (13 October 2020) (Zaveri, 2020), Cleveland Clinic (14 May 2021), Ohio State University (11 June 2024) and Santa Casa de Sao Paulo (Brazil, 11 February 2025).

The two foremost theories of focal epilepsy are the focal theory and network theory. These theories have guided research efforts on epilepsy for the past several decades. The focal theory holds there is pathological activity within a circumscribed cortical area, the seizure focus, such as excessive excitation, or a lack of inhibition, whereby inhibitory control is overwhelmed. This activity then recruits adjacent normal tissue and builds into a seizure. The seizure may evolve further over time before being overwhelmed by inhibitory control and coming to a stop. This inherently plausible and intuitively compelling account of seizure generation has exerted a strong influence in the modern era and remains central to our understanding of epilepsy and to research in the field. A number of factors have been proposed to underlie the aberrance of the seizure focus (for a succinct description, see (Bower, 2024)), but at the heart of this theory is the argument that there exists a circumscribed area which expresses aberrant function, and which is surrounded by normal tissue that can be recruited into the aberrance. While this theory has found support in multiple studies over the past several decades of research, it has also failed to conclusively demonstrate the presence of out-of-control excitation as a path towards seizure generation.

In proposing the network theory of epilepsy, Susan Spencer stated, *“In this context, I consider a network to be a functionally and anatomically connected, bilaterally represented, set of cortical and subcortical brain structures and regions in which activity in any one part affects activity in all the others. The essential operational component of this definition is the observation that vulnerability to seizure activity in any one part of the network is influenced by activity everywhere else in the network, and that the network as a whole is responsible for the clinical and electrographic phenomena that we associate with human seizures. Implicit in this idea is that the seizures may entrain this large neural network from any given part, such that it becomes irrelevant to discuss the “onset” of seizures in any specific part of the network.”* (Spencer, 2002).

The network theory of epilepsy builds on an observed variability of seizure onset but similarity in manifestation of clinical seizures. For example, variable onset of seizures was observed in the hippocampus and entorhinal cortex in patients with medial temporal lobe epilepsy but the seizures were otherwise stereotyped. This is suggestive of activation of the same epilepsy network, though from different anatomic locations (Spencer, 2002; Spencer and Spencer, 1994). The argument for the network theory also drew support from functional neuroimaging and the observation of success with different surgical interventions. The reasoning for the latter was that if different surgical interventions, for example, for medically intractable medial temporal lobe epilepsy, achieved similar levels of seizure control, then each approach must be disrupting parts of the same underlying epilepsy network. Further, the disruption of the different parts of the same network all lead to very similar outcomes for seizure control because a single network was being rendered incapable of generating seizures. The network theory of epilepsy has been influential over the past 2 decades. The network theory, though, has failed to define the nodes and edges of the network involved, what underlies the aberrance of the network and how seizures emerge from a network (Gummadavelli et al., 2018).

In the remainder of the manuscript, we present results of some of our studies and place them within an updated understanding of the network theory of epilepsy. The results we report are based on research performed on the background intracranial electroencephalogram (icEEG) collected from unselected adult subjects undergoing evaluation for possible epilepsy surgery. We study background icEEG, in part, based on the argument that the epilepsy network should be persistently abnormal between seizures and can therefore be defined by the extent and strength of its interictal components and connections (Spencer et al., 2018). Unless indicated otherwise, these studies were performed by collecting background icEEG at two time-points during monitoring, before and after anti-seizure medications (ASMs) were tapered. We recorded 1 h of icEEG, at least 6 h removed from seizures, when the subject was awake, resting and alert at each of these two time-points. The different studies describe temporal, spatial, spectral, and connectivity aspects of the background icEEG. We argue these observations inform an understanding of the network theory of epilepsy by defining the network involved and how seizures are generated within it.

## 2 Observations of the temporal aspects of signal change during icEEG monitoring

Patients are admitted for icEEG monitoring while on ASMs. These medications are subsequently tapered to facilitate the occurrence of seizures. Once a sufficient number of habitual seizures have been collected for surgical decision making, the medications are re-started. In our studies, we considered seizures to correspond to a period of instability. Further, we consider subjects to be relatively stable when on ASMs, and to be relatively unstable after ASM taper. We compared the on-ASMs and off-ASMs time periods to understand the changes that accompany the reduction in stability and give rise to seizures (Spencer et al., 2008; Zaveri et al., 2009a; Zaveri et al., 2010).

We evaluated a large suite of measures of the icEEG from each electrode contact. The measures included a count of interictal spikes and estimates of signal power and power in the delta (0–4 Hz), theta (4–8 Hz), alpha (8–13 Hz), beta (13–25 Hz), gamma (25–55 Hz) and high (65–128 Hz) frequency bands (Spencer et al., 2008; Zaveri et al., 2009a; Zaveri et al., 2010). The measures also included Teager energy, a weighted measure of signal energy which accords higher frequencies with greater energy than lower frequencies (Zaveri, 1993; Zaveri et al., 1993; Kaiser, 1990; Kaiser, 1993).

**FIGURE 1:**
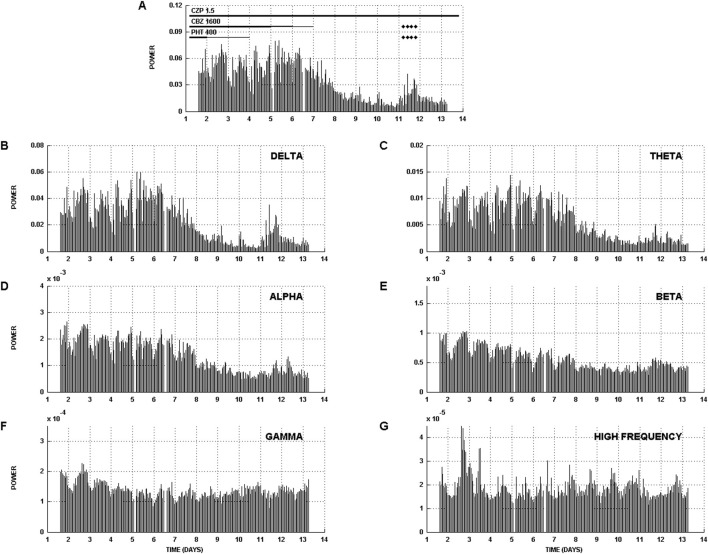
Evaluation of a long-term icEEG study of an example subject with left superior parietal seizure onset evaluated with multiple measures. **(A)** Total power and **(B)** delta, **(C)** theta, **(D)** alpha, **(E)** beta, **(F)** gamma, and **(G)** high frequency band power. Anti-seizure medication (ASM) taper and seizure occurrence is indicated at the top of **(a)**. The units are mV^2^. These results were developed from icEEGs recorded over multiple days and not just on- and off-ASM epochs. The patient was admitted on three ASMs, two of which were tapered. The 3 ASMs were clonazepam (CZP), carbamazepine (CBZ), and phenytoin (PHT). ASM taper is indicated at the top of **(A)** by the thickness of the line drawn next to each ASM. The thickest aspect of each line corresponds to the ASM dose listed. Power values decreased during the course of monitoring to a low point by day 10 and the patient experienced eight seizures during day 11 which are indicated with a marker in **(A)**. Following these seizures ASMs were restarted and the patient was enrolled in a safety study of a neurostimulation device. This resulted in the introduction of noise in the icEEGs, and these data (from days 13 and 14) have not been included in this figure. The corresponding Teager energy and interictal spike count evaluations for this subject can be found in our other reports; see Figure 5 in (Spencer et al., 2008) and Figure 2A in (Zaveri et al., 2009a). The considerable decrease in signal measures, and the emergence of seizures from a state of low power challenge the hypothesis that seizures emerge from out-of-control excitation. Seizures, here, appear to arise from a state of low signal power. Reproduced from (Zaveri et al., 2010).

Our results of the comparison of on- and off-ASM states with these measures were surprising. Most of the broad suite of signal measures demonstrated a decrease with ASM taper. This is illustrated through two figures. In Figure 1, which is reproduced from (Zaveri et al., 2010), we display a multiday evaluation of the icEEG record of an example subject with left superior parietal onset of seizures using total power, and band power in delta, theta, alpha, beta, gamma and high frequency bands. The figure displays hourly estimates of these measures over 13 days of icEEG monitoring. The ASMs and their taper are displayed at the top of Figure 1A. To estimate the power spectral density, hourly icEEG epochs were segmented (segment length 1 s), the mean of each signal segment was subtracted, and the segments were weighted with a Hann window before calculation of the fast Fourier transform. The spectral estimates provided by the Fourier transform were averaged over 1 h. The multiple measures demonstrate a considerable decrease from high values at the start of the monitoring to low values in the latter part of the monitoring. Eight seizures were observed on day 11 of the monitoring, after a considerable decrease in the multiple measurements displayed.

**FIGURE 2:**
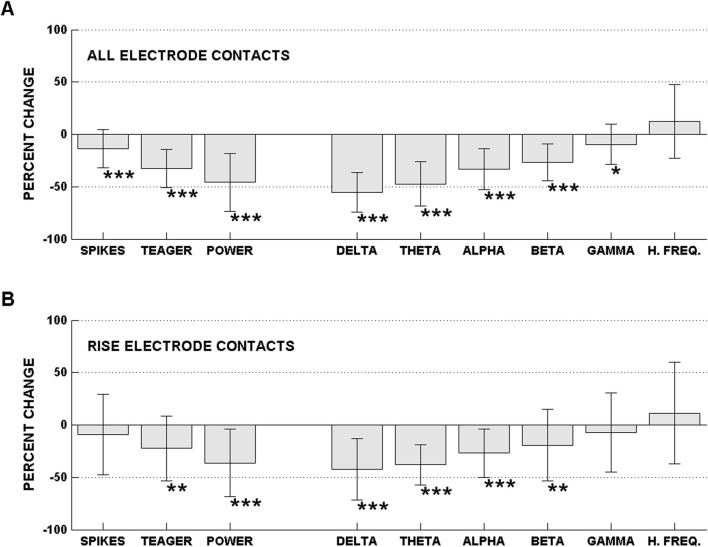
Percent change, between on- and off-ASM states, of spike counts, Teager energy, total power and power of the different frequency bands for **(A)** all electrode contacts and **(B)** region of initial seizure expression (RISE) electrode contacts. Note, in this study, we called the seizure onset area (SOA) the region of initial seizure expression. The two terms SOA and RISE can be used interchangeably. Significant changes are indicated with the following code *** = p < 0.001, ** = p < 0.01, * = p < 0.05. For the all electrode contact measurements there is a decrease in all measures with the exception of high-frequency power. For the RISE (SOA) electrode contacts there is a decrease in all measures with the exception of spike counts and power in the gamma and high frequency bands. The greatest decrease was observed in the lower frequency bands with a diminishing effect with an increase in frequency for both all electrode contacts and RISE contacts. Reproduced from (Zaveri et al., 2010).

In Figure 2, we display the percent change between on- and off-ASM states of interictal spike counts, Teager energy, total power and power of the different frequency bands for all electrode contacts (Figure 2A) and electrode contacts in the seizure onset area (Figure 2B). The results in Figure 2 were obtained from an analysis of icEEG data from 21 subjects. There is a significant decrease in the spike rate, Teager energy, total power and power in the delta through beta bands between the on- and off-ASMs epochs. The greatest decrease was observed in delta band power, with the power in this band decreasing by more than 50% when measured over all electrode contacts. The decrease in the multiple measures was strongly correlated, suggesting a common mechanism underlies the observed signal changes. The decrease in spike counts, for example, was strongly correlated with the decrease in low-frequency power in the delta and theta frequency bands.

We note the background icEEG and results in Figures 1, 2 could have been impacted by the surgery to implant icEEG electrodes. Because of the confound of surgery, we conducted a similar study during scalp EEG monitoring and confirmed a decrease in interictal spikes as measured with scalp EEG accompanied ASM taper (Goncharova et al., 2016).

Figures 1, 2 show a considerable decrease in multiple univariate signal measures between the on-ASMs and off-ASMs periods. This decrease is counterintuitive. Based on this observation, we conclude that seizures do not necessarily arise from a time-period of high interictal activity, Teager energy, signal power or power in different frequency bands. Seizures, rather, can emerge from a time-period with relatively low values of these different measures.

## 3 Observations of low- and high-frequency band-related connectivity of background icEEGs inform surgical outcome

The results described in this and the next section were reported recently (Zaveri et al., 2025). The results are based on a measure of functional connectivity which we call band-related local functional connectivity (BRLFC) (Zaveri et al., 2025). BRLFC is estimated for each icEEG electrode contact and measures the average signal relationship, within a specified frequency band, between that electrode contact and all other electrode contacts in its neighborhood. That is, BRLFC is the average signal relationship, within a frequency band, of a given icEEG electrode contact with all other electrode contacts within a spatial distance, for example, with all other electrode contacts within 5 cm of it. The signal relationship measure we use is magnitude squared coherence which provides us with a measure of signal relationship indexed by frequency and which can be used to determine signal relationship in different frequency bands (Zaveri et al., 1999). BRLFC thus provides a frequency-band-specific and spatially local measure of functional connectivity at each electrode contact. BRLFC was obtained for background icEEG recordings of 14 subjects, from the on- and off-ASMs epochs, for the delta, theta, alpha, beta, gamma and high-frequency bands (Zaveri et al., 2025). The frequency band definitions are as described above. In this manuscript, when referring to our observations on connectivity, we will be referring to connectivity measured by BRLFC.

**FIGURE 3:**
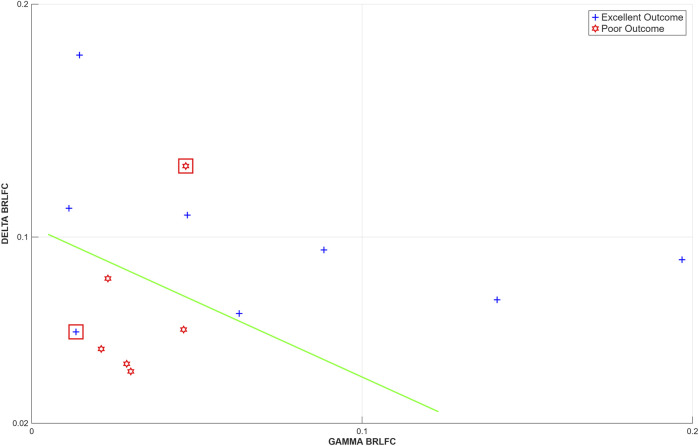
A linear classifier (green line) separates excellent outcome and poor outcome patients based on delta and gamma connectivity in the peri-SOA area. Greater values of delta and gamma connectivity are predictive of excellent outcome while lower values are predictive of poor outcome. One patient each (highlighted) in the excellent outcome and poor outcome groups was misclassified. Reproduced from (Zaveri et al., 2025).

Our evaluation of BRLFC indicated that the primary differences between patients with excellent outcome (EO, Engel’s class 1 or 2, n = 8) and poor outcome (PO, Engel’s class 3 or 4, n = 6) following epilepsy surgery were in the delta, beta, gamma and high-frequency bands (see Figure 3 in (Zaveri et al., 2025)). We observed similarity of BRLFC estimates of alpha through high-frequency bands, as described in the next section, so for this part of the analysis, we focused on one of the high-frequency bands: the gamma band. The highest BRLFC connectivity values in the gamma band were observed in a peri-seizure onset area between 1–5 cm from the SOA. We built a binary classifier based on BRLFC estimates in the delta and gamma bands measured between 1–5 cm from the SOA during the on-ASMs period (see Figure 3). The classifier correctly classified 12/14 patients, misclassifying one patient each in the excellent and poor outcome groups. This result was supported by a leave-one-out analysis performed for this classifier (Zaveri et al., 2025).

The results of the classifier shown in Figure 3 indicate that connectivity in the delta and gamma frequency bands of the background icEEG provides independent information for the classification of subjects as either having an excellent outcome or a poor outcome following epilepsy surgery. Our use of the connectivity estimates of both of these frequency bands contributed to successful classification of excellent and poor outcome patients. While the greatest decreases in univariate measures with ASM taper, discussed in the previous section, were in the lower frequency bands, primarily in delta, the results presented in Figure 3 suggest that there is independent activity in the higher frequency bands of the background icEEG which is also of importance for distinguishing between excellent outcome and poor outcome patients.

## 4 Observations of spatial aspects of the high-frequency connectivity of background icEEGs allow a degree of localization of the seizure onset area

**FIGURE 4:**
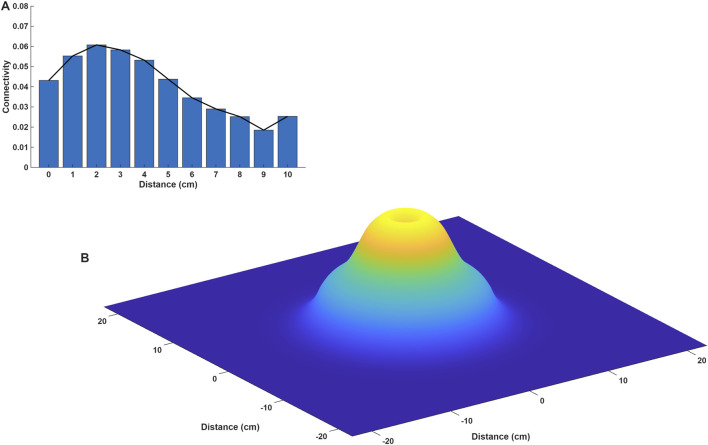
**(A)** Beta band-related local functional connectivity for the on-ASMs period in the excellent outcome group is presented as a function of distance to the SOA. Each value plotted represents the average beta connectivity at that distance from the SOA. **(B)** The results in **(A)** are displayed as a 3D plot. The 3D plot was created by fitting a spline to the 2D profile displayed in **(A)** and rotating it around the ordinate (Y) axis. The 3D plot shows that the connectivity in subjects with excellent outcome has a caldera-like structure, centered around the SOA and with the highest values in peri-SOA areas (1–5 cm from the SOA). In this caldera-like structure the rim of the crater has higher values than the center of the caldera. That is, the highest connectivity is not observed at the SOA. It is observed outside the SOA. There is also a graded relationship between average connectivity and distance to the SOA. This graded relationship can be exploited with a hill climbing search algorithm to localize the SOA as displayed in Figure 5. Modified from (Zaveri et al., 2025).

The BRLFC estimates described in the previous section were also evaluated as a function of the distance of each electrode contact from the SOA (Zaveri et al., 2025). In Figure 4A, we display average beta band BRLFC estimates, for the on-ASMs period, in subjects with excellent outcomes as a function of the distance to the SOA. These values were estimated by converting the distance of each electrode contact to the nearest integer value and averaging the beta band BRLFC estimates of electrode contacts for each integer distance bin. Here, the estimate at distance 0 is the average of the BRLFC estimates of all SOA contacts for all eight subjects with an excellent outcome following surgery. The estimate plotted at a given distance, for example at 1 cm, is the average BRLFC of all electrode contacts at that distance from the SOA, and so on.

The average beta BRLFC of subjects with excellent outcomes in Figure 4A has an intriguing spatial profile with respect to distance from the SOA. In addition to the two-dimensional (2D) plot displayed in Figure 4A we also show this result as a three-dimensional (3D) plot in Figure 4B. The 3D plot was created by rotating the profile in the 2D plot in Figure 4A around the ordinate (Y) axis. The 3D plot shows that the connectivity in excellent outcome patients has a caldera-like structure, centered around the SOA and with highest values in peri-SOA areas. In this structure, the rim of the crater has higher values than the center of the caldera. That is, the highest connectivity is not observed at the SOA. It is observed, rather, in the peri-SOA at distances of 1–5 cm from the SOA. There is also a graded relationship between average connectivity and distance to the SOA. The lowest BRLFC estimates are observed at a distance from the SOA, and the estimates increase as we approach the SOA. We have demonstrated that this BRLFC profile is stable over time, between on- and off-ASMs time-points (Zaveri et al., 2025). While this profile of connectivity as a function of distance to the SOA was clearest in the beta band (shown here), similar profiles were observed from alpha to higher frequencies (Zaveri et al., 2025). This graded relationship was specific to the alpha to higher frequency bands and was not observed in the delta and theta frequency bands (Zaveri et al., 2025).

**FIGURE 5:**
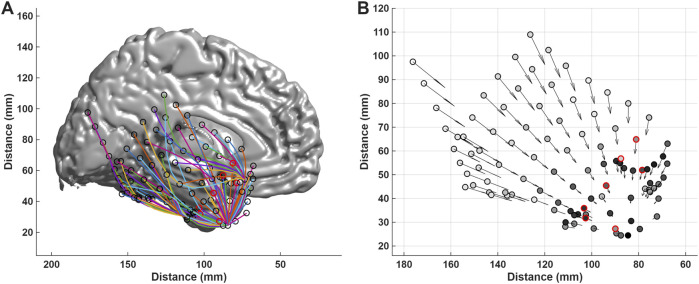
The search paths for localizing the SOA through the use of a hill climbing algorithm on gamma-band-related local functional connectivity. These measurements were performed for a subject with seizure onset in the right anterior superior lateral temporal area. Subdural grid and strip electrodes were placed in this subject to sample parts of the right frontal, temporal, parietal and occipital lobes. The seizure onset contacts in **(A)** and **(B)** are shown with a red outline. **(A)** The search path traversed from each contact is displayed. The search path was developed for each electrode contact on the 3D brain. The right hemisphere was removed from the plot to allow visualization of the different search paths. The color used for each search path is arbitrary. The traced paths from all contacts on the right hemisphere lead to the SOA or to locations near the SOA. **(B)** The resulting search information is displayed as a quiver plot. The vector at each contact has a length proportional to the length of the resultant path of the hill climbing algorithm while the orientation points to the end contact reached for the search from that contact. Each electrode contact is colored with a gray scale proportional to the gamma BRLFC estimate for that contact. The highest BRLFC is observed in the peri-SOA, and the lowest estimates are observed at distances removed from the SOA. The search paths suggest a graded connectivity structure extends from the SOA and peri-SOA to cover a large part of the right hemisphere.

Examples of the BRLFC of individual patients can be found in our previous reports (Zaveri et al., 2009b; Zaveri et al., 2008). The graded connectivity relationship over distance is well defined such that, in some patients, paths can be defined automatically from many of the icEEG contacts to a maximum in the peri-SOA or SOA. That is, we can traverse the connectivity profile from a distant location to a location close to the SOA while blinded to the location of the SOA. We defined a search algorithm to exploit this spatial and spectral structure of connectivity to locate the SOA. The algorithm uses a hill climbing search (Russell and Norvig, 2009) to create a path from each contact in the following manner (Spencer et al., 2018). For a given contact, we examine the connectivity of all contacts within a specified distance of the contact (we first search within 3 cm and if that fails within 5 cm). The search then moves to the electrode contact with the highest connectivity within this distance. Here, the search repeats using the new contact as the center of the search. As the search proceeds, a path is formed from the start contact to an endpoint at an electrode contact with a local or global BRLFC maxima. The paths traced by this search algorithm are illustrated in Figure 5 for a subject with seizure onset in the right anterior superior lateral temporal area and in an evaluation of a patient with two documented SOAs in Figure 3 and Supplementary Figure 1 of our previous review (Spencer et al., 2018).

## 5 An inference based on observations of the temporal, spatial, spectral and connectivity aspects of background icEEG activity

As indicated above, we consider the off-ASMs state to be a time-period of relative instability as seizures are more likely to arise from this state. This is also, during icEEG monitoring, a time-period when the subject is relatively closest to their native state for the following reasons. First, it is a time when ASMs have been tapered. Second, it is several days after surgery to implant electrodes. Third, it is after the first few days in the epilepsy monitoring unit when the subject may not sleep well (Joshi et al., 2016). The observations presented in Figures 1, 2 describe the changes which occur with the transition from an on- to off-ASMs state. The observations presented are counterintuitive in that they demonstrate a considerable decrease in a diverse set of measures of icEEG activity, including interictal epileptiform activity, energy, signal power, and band power from the on- to off-ASMs state. This temporal pattern is difficult to reconcile with the idea that seizures arise from excess or unregulated excitation, as proposed by the focal theory of epilepsy.

Our observations suggest that seizures can arise from a state of decreased cortical activity. In reference to our observations of the changes between on- and off-ASM states, we stated the following in our previous publication (Zaveri et al., 2010): *“The observations are intriguing because seizure, a hyperexcitable state, arises from this state of decreased cortical icEEG activity. It is important to incorporate this information within a theory of seizure initiation and we hypothesize the following. We note that the large decrease in cortical tone between on- to off-AEDs states will result in a concomitant decrease in feedback to thalamic neurons through cortico-thalamic pathways. Such a decrease may result in a change in thalamic activity from tonic to burst mode. In burst mode thalamic neurons can more powerfully evoke cortical activity and thalamic burst firing may serve to increase cortical synchronization* (Briggs and Usrey, 2008; Swadlow and Gusev, 2001; Sherman, 2001)*. The increase in high frequency activity observed in* (Zijlmans et al., 2009) *and in some patients here may reflect such a change. A change from tonic to burst mode in thalamic nuclei and a resultant increase in cortical activity and synchronization could lead to seizure. While the results presented here support only part of this theory, and the reminder remains speculative or partially supported* (Hodaie et al., 2006) *this theory could serve as a basis for the design of experiments to test the involvement of cortico-thalamic activity and the thalamus in the initiation of seizures in localization related epilepsy.”*

Our argument, therefore, is that our observations suggest reduced or failed signaling from the cortex to the thalamus. This may trigger an interaction cascade: when the thalamus does not receive adequate cortical input, it responds by attempting to “wake up” the cortex.

The temporal profile shown in Figure 1 was the most common profile we observed: one where there is a decrease, which is followed by seizures. Other profiles were also observed, including where seizures occurred on the upslope, downslope or at times of relative maxima (Spencer et al., 2008; Zaveri et al., 2009a; Zaveri et al., 2010). Some of these are reminiscent of features observed in multiday cycles in RNS feature series (Baud et al., 2018). The most common change we observed, though, was the occurrence of seizures following a decrease in signal measures and with maximal decrease being observed in the delta band as shown in Figures 1, 2. In related work, we have shown that while delta power and multiple univariate signal measures decrease with ASM taper, the envelope coherence of the delta power time-series increases between the on- and off-ASM time periods and in time-periods around seizures (Joshi et al., 2018). We have suggested that this increase in the envelope coherence of the delta power time-series could reflect a priming process that leaves the patient more susceptible to seizure generation and propagation (Joshi et al., 2018). Additionally, a recent study lends support to the involvement of the thalamus in the initiation of seizures (Ilyas et al., 2023; Wiebe and Gotman, 2023). In this study, Ilyas and co-workers subjected icEEG recordings from the seizure onset zone and the thalamus to a deep neural network and demonstrated successful detection of a pro-ictal period either 35 or 45 min prior to seizure (Ilyas et al., 2023).

When considering the connectivity results, we would like to draw attention to two observations. First, we found both low (delta) and high frequency (gamma) BRLFC estimates to be useful in the classification of excellent and poor outcome patients. While high BRLFC estimates in delta and gamma bands are each indicative of an excellent outcome following epilepsy surgery, their conjunction results in the classification accuracy that was obtained. Second, the spatial profile of high-frequency connectivity presents a graded structure with respect to distance from the SOA. This spatial structure extends several cm from the SOA and is not observed in the low (delta and theta) frequencies (Zaveri et al., 2025). In our interpretation, this suggests a cortico-cortical relationship with the SOA exists in higher-frequencies and not in lower frequencies.

**FIGURE 6:**
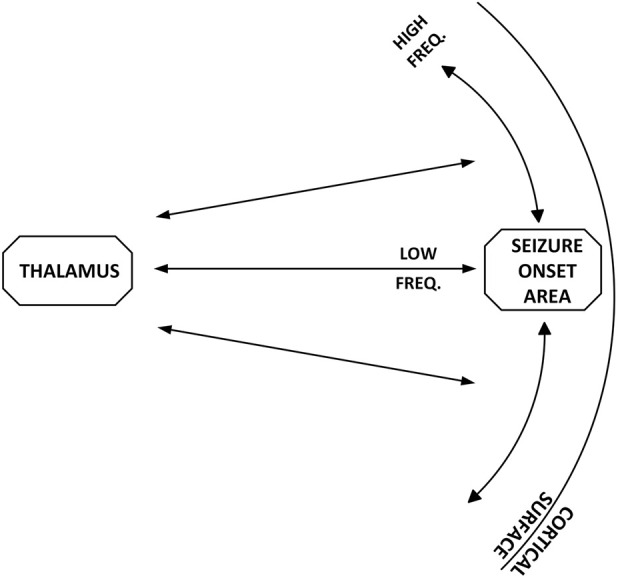
We suggest the presence of two nodes in the seizure-generating network, the SOA and a sub-cortical structure, the thalamus. The activity of the different parts of this network is reflected differently in the icEEG. The first is a low-frequency component with activity primarily reflected in the delta band, which is suggestive of thalamo–cortical and cortico–thalamic interactions, and which we propose plays a central role in seizure initiation. The second is a high-frequency component suggestive of cortico–cortical interaction and which we propose plays a role in seizure spread. This figure is adapted from (Zaveri, 1993).

While we caution there are limitations to the primary studies which form the basis of the summary provided here (Spencer et al., 2008; Zaveri et al., 2009a; Zaveri et al., 2010; Zaveri et al., 2025), in that they were all conducted on data acquired during icEEG monitoring and the number of subjects studied was small. Based on the temporal, spatial, spectral and connectivity results we believe we are, in the main, observing two separate phenomena in the background icEEG. First, low-frequency activity in the delta band, that can decrease profoundly with ASM taper, and where seizures occur when signal power estimates are low. The connectivity in this band does not suggest cortico-cortical relationship. Second, high-frequency activity (alpha and higher), that in contrast, demonstrates a cortico-cortical profile related to distance to the SOA. Based on the temporal, spatial, spectral and connectivity observations, we propose a two-node model of the seizure-generating network. One node corresponds to the SOA. The second node we propose is a sub-cortical structure, the thalamus. While the first node, the SOA, has been studied considerably, the second node, the thalamus, has only recently started to be investigated in focal epilepsy. A schematic of the model is displayed in Figure 6. The activity of the different parts of this network is reflected differently in cortical recordings. The first is a low-frequency component, reflected in the delta band, suggestive of the relationship between the thalamus and cortex and which may play a central role in seizure initiation (Zaveri et al., 2010; Joshi et al., 2018). The second is a high-frequency component suggestive of a cortico-cortical relationship, which may reflect seizure spread. Note, while we suggest that the high-frequency component reflects cortico-cortical spread of seizure activity, we do not preclude the possibility that seizure propagation may also occur through cortico-thalamic and thalamo-cortical projections.

We acknowledge that additional cortical and subcortical regions may participate in seizures. The cortex forms reciprocal circuits with the thalamus, basal ganglia, and cerebellum, creating parallel systems that modulate cortical activity. The basal ganglia—particularly in animal studies—have been shown to exert modulatory effects in both absence and temporal lobe seizures (Vuong et al., 2018). Other potentially involved regions include homologous cortical areas. However, these areas and structures are unlikely to serve a primary role comparable to the central function we hypothesize for the thalamus. They may play a modulatory role.

## 6 Tonic and burst firing of thalamic neurons

In Exploring Thalamocortical Interactions: Circuitry for Sensation, Action, and Cognition, W. Martin Usrey and S Murray Sherman state, *“… neither that thalamus nor the cortex can be understood in any meaningful way in isolation of the other. Rather, proper function of cortex and thalamus requires thalamocortical interactions, mediated by thalamocortical and corticothalamic projections, each of which includes multiple pathways with specific anatomical and physiological properties*” (Sherman and Usrey, 2021).

Within large-scale brain networks, the thalamus occupies a uniquely central position. It is distinguished by its dense and reciprocal connectivity with the cerebral cortex: virtually every cortical region receives input from one or more thalamic nuclei, and each thalamic nucleus, in turn, is extensively innervated by the cortex. This bidirectional architecture places the thalamus at the core of cortical communication and coordination. Particularly critical are the dynamic interactions among thalamocortical relay neurons, thalamic reticular neurons, and corticothalamic neurons, whose tightly coupled activity underpins thalamocortical network function. Of importance also are brainstem cholinergic neurons, which modulate the activity of thalamocortical relay neurons and thalamic reticular neurons. Thalamocortical relay and reticular neurons fire in two modes, in tonic mode or burst mode, with the latter resulting from a hyperpolarization of the cell membrane. Tonic firing by thalamocortical relay neurons communicates information to the cortex with high fidelity while burst firing underscores the saliency of the information being relayed. Although burst firing by thalamocortical relay neurons conveys less precise information about stimulus features, it is particularly effective at driving cortical responses and enhancing signal detectability (Steriade et al., 1997). One of the theories posited is that burst firing of thalamocortical relay neurons serves to wake up the cortex (Sherman, 2001; Swadlow and Gusev, 2001). Burst firing by thalamic reticular neurons is inhibitory and primarily serves to synchronize and gate thalamocortical relay activity, thereby indirectly shaping cortical responsiveness.

## 7 The two-node network hypothesis and its implications

The initial statement on the network theory of epilepsy was informed by visual analysis of the icEEG, primarily seizures; clinical information on the patients being studied; neuroimaging and epilepsy surgery (Spencer, 2002). It has provided a very influential general perspective on the involvement of large-scale networks in epilepsy. While a few example networks were described in the paper, other than the limbic network, these networks have not proven to be common. A network, at a minimum, is composed of two nodes and directed edges between these nodes (Gummadavelli et al., 2018). The initial statement did not provide clear information on the nodes and edges of the networks involved. Further, it did not define the mechanisms underlying seizure initiation and termination. There were, thus, shortcomings to the network theory as initially stated.

We have presented a synopsis of our published results on temporal evolution, spatial structure, spectral structure and connectivity of background icEEGs. These observations taken together are used to propose a hypothesis on the network theory of epilepsy, including aspects which were missing in the initial statement. The implications of our hypothesis are far reaching and may hold meaning for central aspects of our understanding of epilepsy, including those which pertain to seizure initiation, propagation, termination and control. The following are key aspects of our hypothesis and its implications.

First, we hypothesize a two-node network, where one node is the SOA and the second is the thalamus. That is, the network is the SOA and the parts of the thalamus that are connected to it. The network edges, the connections between the two nodes, are thalamocortical and cortico-thalamic projections. The network, thus, is not novel but extant with established connections (Zaveri et al., 2020). While we define the network to be composed of two nodes and the interaction between them, we do not mean this to be limiting. The SOA does interact with cortical areas that it is connected to, and these cortical areas could be considered to be one or more additional nodes. We note that we have described nodes, here, in a general manner. The nodes may have nodes or sub-networks within them, and they are likely connected to other areas and networks that influence their behavior, for example, the brainstem as mentioned in the previous section. In the main though, we hypothesize that focal epilepsy results from a two-node network, composed of the SOA and the thalamus.

Second, we propose seizures arise from the interaction between these two nodes. Seizure initiation may result because the thalamus has not received adequate input from the cortex, triggering burst firing in the thalamus. Insufficient input may result from cortical injury, a characteristic of many animal models of epilepsy. This suggests a central role in seizure initiation for the long-established observation of hypometabolism in the SOA by fluorodeoxyglucose positron emission tomography (FDG PET) studies (Juhasz and Chugani, 2003; Csaba, 2003; Vinton et al., 2007) and decreased mitochondrial activity described by quantitative magnetic resonance spectroscopic imaging (MRSI) (Hetherington et al., 2007; Pan et al., 2008; Pan et al., 2013). Our argument is that attenuated activity in the SOA triggers burst firing in the thalamus. This, in turn, suggests that seizure termination may occur because the heightened cortical activity during seizure meets the input requirements of the thalamus. Part of this sequence of activity is reminiscent, in a general manner, of the suggestion that seizures correspond to a resetting mechanism (Iasemidis et al., 2004), though that description was developed for the interaction between the SOA and normal cortex and not the thalamus. The argument made here may also suggest status epilepticus is a phenomenon where the triggered seizure does not meet the cortical input requirement of the thalamus, thus resulting in continually triggered seizures. Separately, we note that during rapid eye movement (REM) sleep there is a suppression of thalamic burst firing. The attenuation of thalamic burst firing in REM sleep could explain the known low likelihood of seizure occurrence during REM (Ng and Pavlova, 2013). An important implication here is that focal seizures are a normal, though extreme, part of the interaction between the thalamus and cortex.

Third, there are implications for surgical treatment of epilepsy. First, the suggestion is that the thalamus is a missing part of the network which we need to monitor and study. We will need to better understand the role of the thalamus in seizure initiation, propagation and termination. If our hypothesis is proven correct, studying just the SOA, as we have done historically, could be akin to trying to understand how two hands produce a clap by analyzing the activity of a single hand. To test the proposed hypothesis, both nodes of the network will have to be sampled. The thalamus and SOA should be sampled in a manner that allows capture of interactions between the two, rather than sampling two locations which may or may not be connected to each other. Additionally, attention must be paid to the modality used to sample the two nodes. It may not be the case that both should be sampled with the same modality to observe their interaction. Second, our hypothesis has implications for neuromodulation for seizure control. In accordance with our hypothesis, the goal of neuromodulation should be to produce cortical output to meet the input requirements of the thalamus. If cortical activity can be modulated to provide sufficient input to the thalamus, this may reduce the drive for seizure initiation. That is, the cortex must express sufficient activity as feedback to the thalamus to satisfy the input requirements of the thalamus and in this manner reduce the vulnerability to seizures.

Fourth, thalamocortical interactions are fundamentally implicated in absence epilepsy. The coordinated interplay among thalamocortical relay neurons, thalamic reticular neurons, and cortical neurons—particularly through burst firing—is thought to generate the characteristic spike-and-wave discharges seen in absence seizures. The conceptual overlap between this framework and the hypothesis advanced here raises the possibility of a unified, consolidated theory of epilepsy grounded in thalamocortical dynamics. The value of a unifying theory, spanning all epilepsies, cannot be overstated. Consistent with this reasoning, extensive work on spike-and-wave seizures suggests that the EEG spikes in absence epilepsy may reflect synchronous neuronal firing orchestrated by thalamic circuits. This principle of thalamically mediated synchrony may also provide important insight into the generation of EEG spikes in focal epilepsy. Our observation of a correlated decrease in icEEG spike counts and power in the low frequency (delta and theta) bands supports this line of reasoning.

Finally, we suggest that our findings and this two-node network describe a model for the EEG. The interaction between the thalamus and cortex is reflected in a low-frequency band, primarily the delta band. The interaction between the SOA and adjacent cortex is reflected primarily in a higher frequency band, which ranges from alpha to higher frequencies, with the clearest reflection of cortico-cortical interactions being in the beta and gamma frequency bands. This decomposition of the EEG into low-frequency (delta, theta) and high-frequency (alpha and higher) bands may have value for understanding the normal EEG.

## 8 Conclusion

We have proposed a two-node interaction theory for focal epilepsy. This theory defines the two nodes as the SOA and thalamus and postulates that the interaction between them can give rise to focal seizures.

## Data Availability

The data analyzed in this study is subject to the following licenses/restrictions: These data cannot be shared without approval of the Yale University Human Investigation Committee and funds for data curation and sharing. Requests to access these datasets should be directed to hitten.zaveri@yale.edu.
